# Development of a multiphase perfusion model for biomimetic reduced-order dense tumors

**DOI:** 10.1007/s42757-022-0150-x

**Published:** 2023-03-05

**Authors:** Mohammad Mehedi Hasan Akash, Nilotpal Chakraborty, Jiyan Mohammad, Katie Reindl, Saikat Basu

**Affiliations:** 1. Department of Mechanical Engineering, South Dakota State University, Brookings, SD 57007, USA; 2. Department of Biomedical Engineering and Mechanics, Virginia Tech, Blacksburg, VA 24061, USA; 3. Center for Diagnostic and Therapeutic Strategies in Pancreatic Cancer, North Dakota State University, Fargo, ND 58108, USA; 4. Department of Biological Sciences, North Dakota State University, Fargo, ND 58108, USA

**Keywords:** solid tumor, multiphase simulation, plasma perfusion, computational modeling, biomimetic analysis

## Abstract

Dense fibrous extracellular constitution of solid tumors exerts high resistance to diffusive transport into it; additionally, the scarcity of blood and lymphatic flows hinders convection. The complexity of fluidic transport mechanisms in such tumor environments still presents open questions with translational end goals. For example, clinical diagnosis and targeted drug delivery platforms for such dense tumors can ideally benefit from a quantitative framework on plasma uptake into the tumor. In this study, we present a computational model for physical parameters that may influence blood percolation and penetration into simple biomimetic solid tumor geometry. The model implements three-phase viscous-laminar transient simulation to mimic the transport physics inside a tumor-adhering blood vessel and measures the constituent volume fractions of the three considered phases, viz. plasma, RBCs (red blood cells, also known as “erythrocytes”), and WBCs (white blood cells, also known as “leukocytes”) at three different flow times, while simultaneously recording the plasma pressure and velocity at the entry point to the tumor’s extracellular space. Subsequently, to quantify plasma perfusion within the tumor zone, we proposed a reduced-order two-dimensional transport model for the tumor entry zone and its extracellular space for three different fenestra diameters: 0.1, 0.3, and 0.5 μm; the simulations were two-phase viscous-laminar transient. The findings support the hypothesis that plasma percolation into the tumor is proportional to the leakiness modulated by the size of fenestra openings, and the rate of percolation decays with the diffusion distance.

## 1 Introduction

Perfusion mechanics into a dense solid tumor has long been recognized as a critical issue in both clinical and experimental studies (Gullino, 1980; Vaupel et al., 1990; Jain, 1996). Owing to the constriction of tumor blood vessels and abnormal leakiness into the tumor extracellular space from the tumor vasculature, blood perfusion into tumors can often be much lower than in normal tissues surrounding them (Stylianopoulos and Jain, 2013). Solid tumor constitutions are seen in the majority (~90%) of tumor cases that can occur throughout the body. A solid tumor presents an unusual mass of tissues that does not contain cysts or liquid regions, and can be categorized as benign (non-cancerous) or malignant (cancerous), see, e.g., Gavhane et al. (2011). These tumors exhibit high resistance to diffusive transport (Jain, 1996, 1998), and the scarcity of blood and lymphatic flows in the surrounding environment impedes convection. These nuances in flow physics render it difficult for blood to perfuse into the tumor volume (Chim and Mikos, 2018), despite the increased permeability and retention effects frequently observed during nanoparticle delivery to tumor tissues (Maeda, 2012). Accurate quantification of the spatio-temporal distribution of particulates in the blood channel and tumor region could be critical for cancer clinical diagnosis (d’Esposito et al., 2018). The development, spread, and treatment of cancer all have a connection to fluid mechanics. Tumor growth and drug delivery are governed by multi-scale flow–structure interaction processes, while the interstitial pressure in the tumor microenvironment is accentuated by the tumor vasculature’s irregularity and leakiness (Koumoutsakos et al., 2013). However, the pathophysiology of diseased tissues interacts chaotically with the blood uptake process (Soltani and Chen, 2011), and on a macro-scale, can vary between subjects resulting from the differences in tumor topography (Zhang et al., 2013). Review of the biomedical science literature demonstrates that single-phase computational fluid dynamics (CFD) models, which treat blood as a single continuum of homogeneous fluid (Womersley, 1957; Attinger, 1964), cannot provide the hemodynamic information necessary to quantify the interactive effects and spatial distribution of suspended particulate matters in blood (Srivastava, 2007). In practice, blood exhibits a non-Newtonian rheology (Merrill et al., 1963) owing to the complex strain rates as well as high cell count and particulate nature of RBCs (red blood cells). For concentrated suspensions (Baskurt and Meiselman, 2003), the spatial variation of blood constituents such as plasma, RBCs, and WBCs (white blood cells) may vary owing to the disturbed flow within the tumor region. RBCs collide with one another in such flows as a result of their relative motion and can aggregate to form larger RBC agglomerates. This phenomenon, referred to as RBC aggregation, is merely one of the inter-particle phenomena of blood flow that affects the rheological behavior of blood and thus the ambient distribution of particulate matter around the solid tumor. As a result, developing a more nuanced CFD model is critical to realistically mimicking the multiphase fluidic transport in the blood vessels adhering to the connective tissues in solid tumors.

While perfusion into the tumor has been identified as a significant issue for cancer research, herein we attempt to answer the following hypothesis-driven question: can we spot the impact of tumor leakiness on plasma perfusion into the tumor via our reduced-order modeling approach with a two-dimensional (2D) biomimetic tumor domain through considering different sizes of fenestra openings on the tumor vasculature?

To answer the aforementioned, we used CFD techniques to model the blood transport process in the generated biomimetic model. This study’s preliminary findings were presented at the American Physical Society’s Division of Fluid Dynamics Annual Meeting 2021 (Akash et al., 2021).

## 2 Methods

### 2.1 Development of a biomimetic tumor microenvironment model

**Fig. 1:**
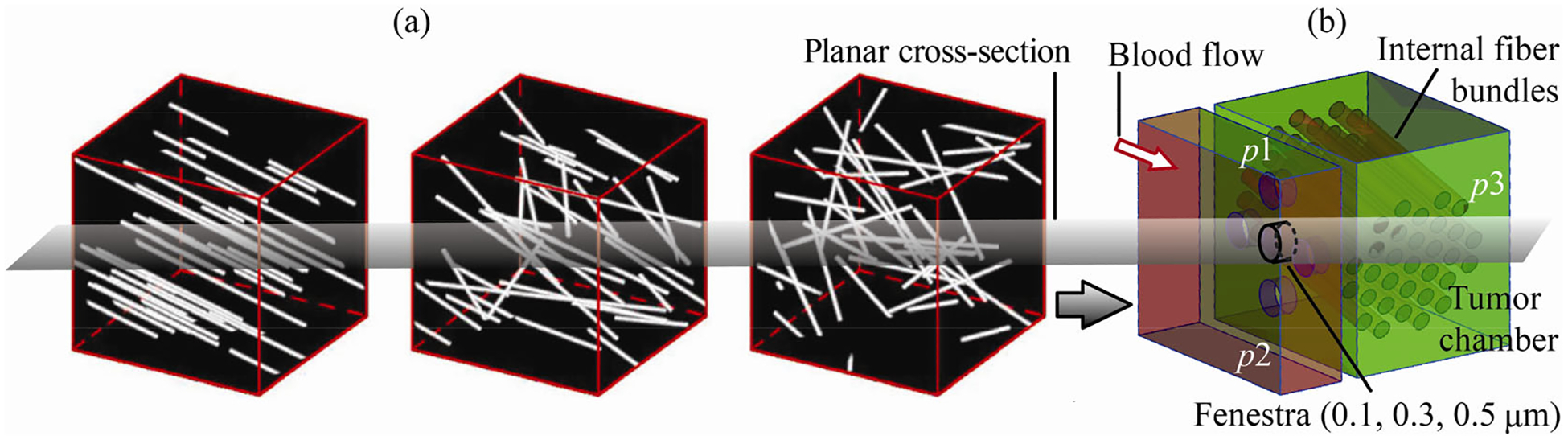
(a) Extracellular fiber matrix in scanned tumor domains (reproduced with permission from Eekhoff and Lake (2020), © The Author(s) 2020). (b) Our preliminary 3D biomimetic concept with random fiber orientation. Information on length scales: for (a), the fiber persistence lengths are on average 20 μm, as per Eekhoff and Lake (2020) and Stein et al. (2008). Also note that (b) is for spatial conceptualization of the developed model and is not to scale. Here, p1=3325Pa,p2=2128Pa, and p3=2780Pa (see Section 2.4 for details).

**Fig. 2:**
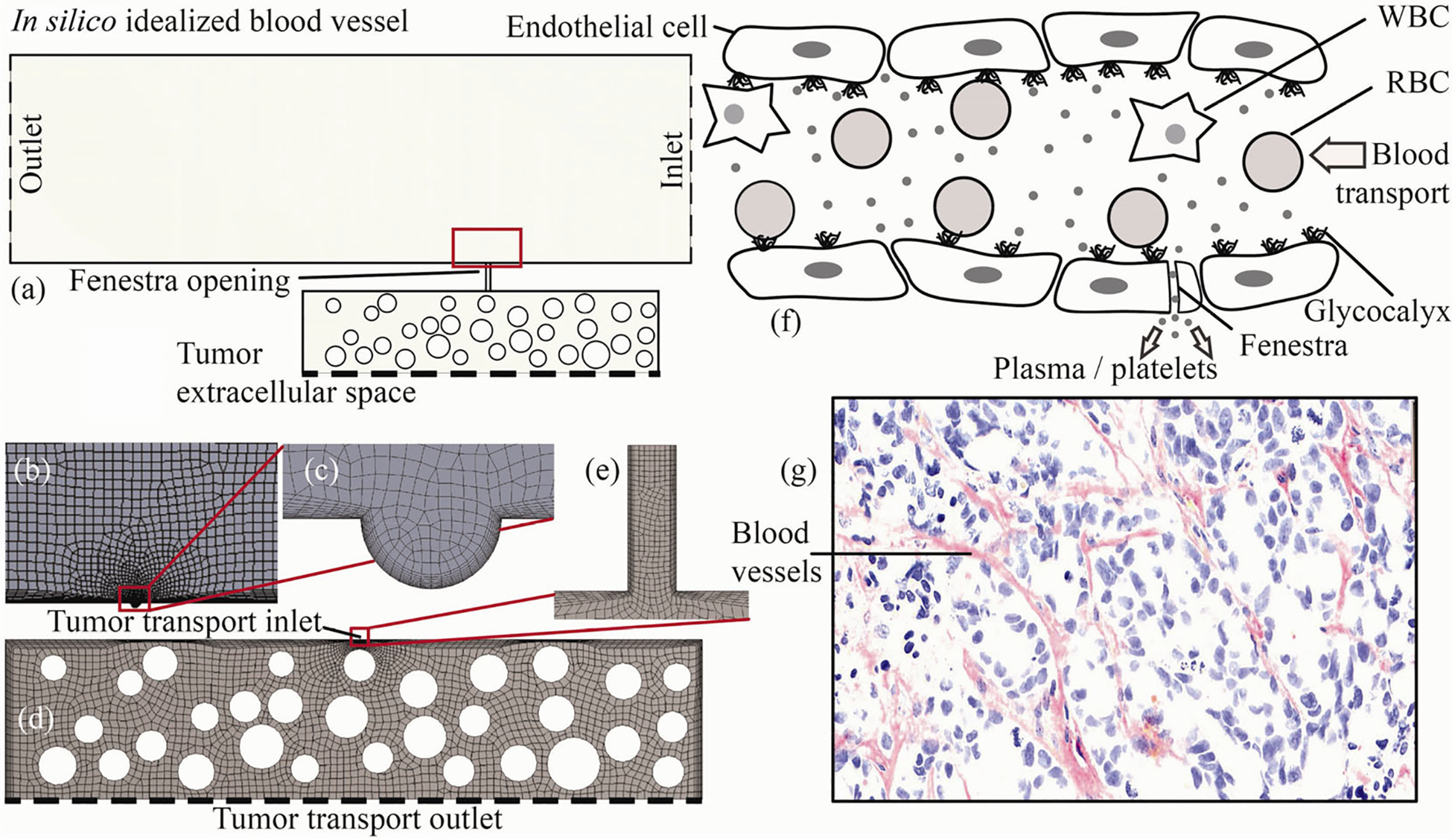
(a) 2D biomimetic domain for a tumor microenvironment, where the blood vessel is connected with the tumor stroma via a fenestra. To facilitate numerical experiments, the space in (a) is then segregated into two segments. The strategy restricts the entry of the particulate matter suspended in blood inside the fenestra whose diameter is smaller than those particle sizes. (b–e) Two segments of the test geometry with the corresponding mesh elements. Therein, (b) shows a part of the blood vessel with a circular depression that coincides with the location of the fenestra opening marked in panel (a); (c) zooms in on the artificial depression; (d) shows the tumor region with fenestra; and (e) depicts the fenestra space with finer mesh elements. The dashed bottom lines in (a) and (d) depict the outlet sink for tracking the intra-tumoral uptake. The simulation of the multiphase transport process in the blood vessel generates the local pressure and velocity of plasma at the artificial circular depression. The data are relayed and the dynamics of the same plasma volume fraction is then tracked inside the tumor interiors in the subsequent step of the numerical simulations that use the tumor extracellular matrix, i.e., (d), as the test geometry. (f, g) Sample views of the realistic systems, respectively that of a blood vessel in the tumor vasculature (in the form of an artistic sketch) and a scanned cross-section through a dense pancreatic tumor implanted in a mice. The views serve as the biomimetic inspiration for the two main segments of our reduced model (i.e., the blood vessel and the tumor stroma). Note that (f) and (g) are on different length scales. In (g), the resolution of this image is 20×.

The extracellular matrix in a tumor microenvironment supports the biological tissues mechanically and the fiber alignment influences cellular behavior (Eekhoff and Lake, 2020). We first conceptualized a three-dimensional (3D) spatial domain for a blood vessel with adjoining connective tissues of a solid tumor. The conceptualization is based off published computed tomographic reconstructions (Fig. 1(a)). A subsequent 2D slice through the 3D idealization (Fig. 1(b)) guided the formation of the *in silico* biomimetic geometry (Fig. 2(a)) used in this study.

The spatial orientation of the preliminary 3D model’s internal fiber bundles was determined using Fig. 1(a) (Eekhoff and Lake, 2020). The subsequent 2D biomimetic section through the 3D space was built on the Workbench 2019 R3 New Design Modeler Geometry (DM) (ANSYS Inc., Canonsburg, Pennsylvania, USA). We next segregated this model (Fig. 2(a)) into two parts to restrict the entry of bigger particles into the smaller path, e.g., to restrict the entry of RBCs (with diameters of 7–8 μm, see Nithiarasu (2022)) into the fenestra (with its maximum diameter in our model being 0.5 μm). The two segregated parts are: (i) the blood vessel with a micron-scale rounded depression signifying the entry location to the tumor extracellular space; and (ii) the tumor extracellular domain with a fenestra opening. The depth of the depression was 2.6 μm, and the diameter was 5.2 μm. The depression depth was chosen such that it is larger than 8 times the height of a fenestra’s streamwise length (which was 0.3 μm), in order to ensure that the entire fenestra space is contained within the depression region, which is critical for the biomimetic realism of the system with the fenestra serving as the link between the blood vessel and the tumor region. With a similar rationale, the diameter of the circular depression is designed to be bigger than 10-fold of that of the maximum fenestra diameter (0.5 μm) in the biomimetic model. The zoomed views of the depression in the blood vessel are shown in Figs. 2(b) and 2(c). Figure 2(d) depicts the tumor domain with fenestra. We modeled three distinct tumor domain geometries using three different yet realistic fenestra diameters of 0.1 μm (Model 1), 0.3 μm (Model 2), and 0.5 μm (Model 3), with the streamwise fenestra length remaining constant at 0.3 μm in each case. Figures 2(b)–2(e) show the finer mesh elements, which were generated on ANSYS Workbench 2019 R3 Mesh. Each computational grid in this study is highly resolved with more than 0.02 million unstructured and graded octahedral components, with 0.04 million for blood vessel (Fig. 2(b)), and 0.023 million for the tumor geometry (Fig. 2(d)).

Figure 2(f) conceptualizes the multiphase nature of transport considering RBCs, WBCs, and plasma as the dominant constituent phases in a blood vessel in the tumor microenvironment, with the fenestra allowing extravasations of the plasma components. For the lower half of the segregated domain in Fig. 2(a), i.e., for the tumor geometry, the spatial coverage of the fiber bundles was carefully determined such that the packing fraction (calculated as the ratio of the fiber cross-sections over the tumor area) stays consistent with the corresponding measurements in real tumors, the same quantity also being a marker of the solid stress in such dense tumors. As a specific example, the mice tumor cross-section in Fig. 2(g) was analyzed for color decomposition to return a mean packing fraction of 0.30, with the value ranging between 0.23 and 0.41 locally in discrete isolated regions of the scan. In comparison, the packing fraction in our biomimetic model was 0.27.

### 2.2 Simulation of blood transport inside the blood vessel

The viscous-laminar transient state flow physics dominates the *in silico* transport inside blood vessels. To numerically track the process, we used the commercial software package ANSYS FLUENT 2019 R3, which includes a 3D Eulerian multiphase model without the need for custom inter-particle equations. Our model of blood transport is composed of RBCs and WBCs suspended in plasma as two scattered phases. The framework is an extension of Gidaspow’s (1994) and Anderson and Jackson’s (1967) multiphase CFD approach. This method is particularly effective for modeling blood flows with closely-spaced RBCs, presenting a volume fraction between 30% and 55% *in vivo* (Jung et al., 2006). The multiphase system, as compared to a single-phase model, also critically incorporates the volume fraction of each phase as well as the momentum exchange mechanism between the phases. We developed user-defined functions for the shear thinning viscosity of RBCs and the effect of RBC agglomeration using equations from Jung and Hassanein (2008). The effective RBC shear viscosity ($\eta_{\text{RBC}}$) was calculated from the equation for dimensionless relative blood mixture viscosity ($\mu_{\text{mix}}$) in a non-Newtonian shear-thinning fluid:

(1) $$\mu_{\text{mix}}=\frac{\Psi_{\text{RBC}}\eta_{\text{RBC}}+\Psi_{\text{plasma}}\eta_{\text{plasma}}+\Psi_{\text{WBC}}\eta_{\text{WBC}}}{\eta_{\text{plasma}}}\;=a{\left[1+{\left(\tau \dot{\gamma }\right)}^{2}\right]}^{\frac{b-1}{2}}$$

Here, $\dot{\gamma }$ is the shear rate (1/s). The volume fraction and viscosity are represented by Ψ and η, respectively. The time constant τ was set to 0.11 s, and the two parameters a and b as functions of the hematocrit were given by the polynomial approximations for $\dot{\gamma }$ greater than or equal to 6 (Jung et al., 2006). Note here that the hematocrit ($\Psi_{\text{RBC}}$) is defined as the ratio of the volume of RBCs to the total volume of blood in the vessel.

$$a=122.28\Psi_{\text{RBC}}^{3}-51.213\Psi_{\text{RBC}}^{2}+16.305\Psi_{\text{RBC}}+1$$

$$b=0.8092\Psi_{\text{RBC}}^{3}-0.8246\Psi_{\text{RBC}}^{2}-0.3503\Psi_{\text{RBC}}+1$$

For $\dot{\gamma }$ less than 6 at low shear rates, these were as

$$a=70.782\Psi_{\text{RBC}}^{3}-22.454\Psi_{\text{RBC}}^{2}+9.7193\Psi_{\text{RBC}}+1$$

$$\frac{b-1}{k}=-0.8913\Psi_{\text{RBC}}^{3}+2.0679\Psi_{\text{RBC}}^{2}-1.7814\Psi_{\text{RBC}}$$

where $k=\frac{\text{ln}\;(\text{ln}\;\dot{\gamma })}{\text{ln}\;\dot{\gamma }}$.

**Fig. 3:**
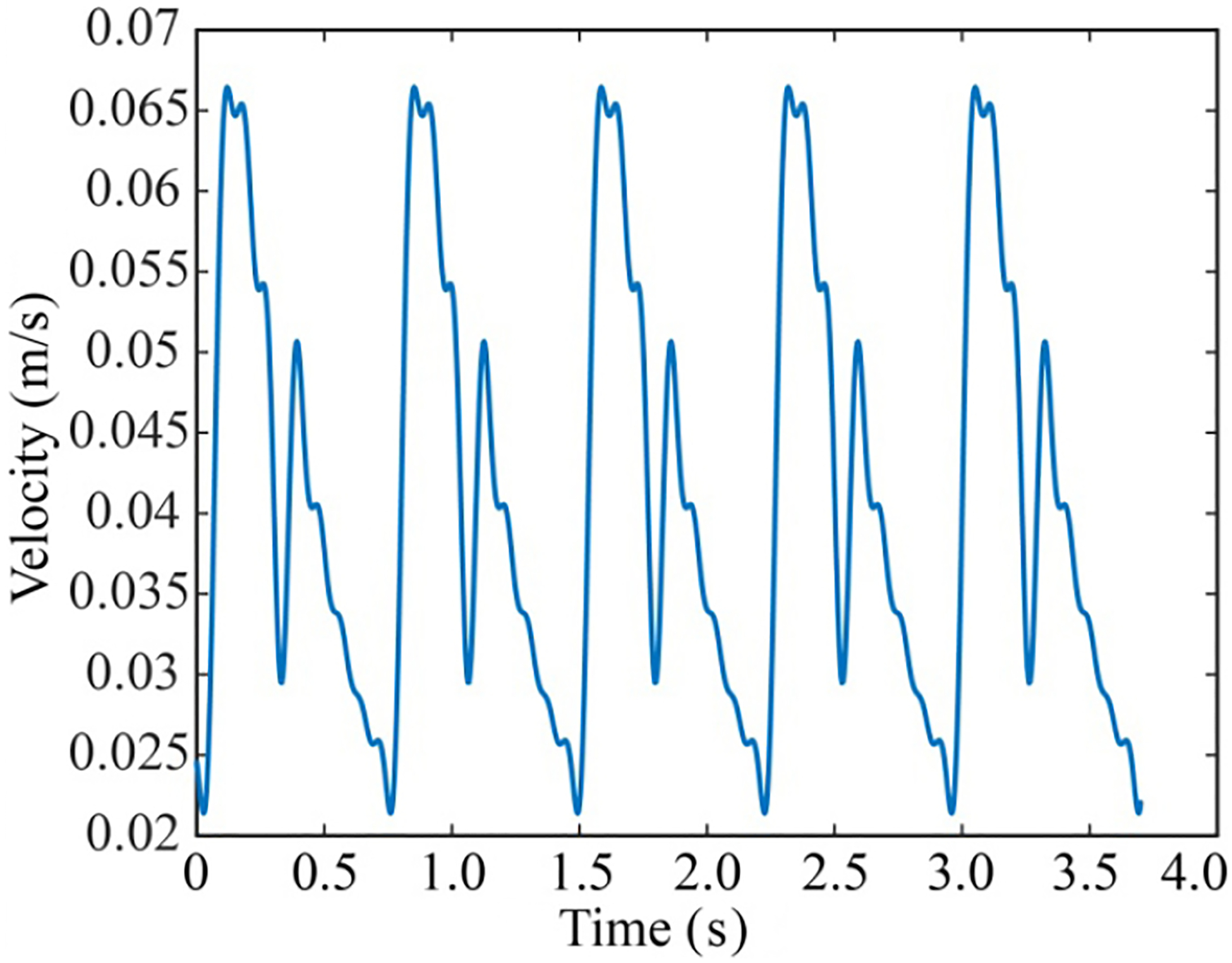
Inlet velocity trend in the blood vessel corresponding to an actual heart cycle. The periodic profile was generated by mapping a Fourier transform from previously published experimental data (Cholley et al., 1995; Poppas et al., 1997; Vlachopoulos et al., 1998).

The inlet velocity is pulsatile with a time period of 0.735 s (Fig. 3). To match it with an actual heart cycle, published experimental data (Cholley et al., 1995; Poppas et al., 1997; Vlachopoulos et al., 1998) are mapped into a Fourier transform with MATLAB curve fitting tool. Both RBCs and WBCs experience interphase drag from plasma, which is a type of fluid–particle interaction. This interaction was described using the interphase momentum exchange coefficient α, and the velocity differences between the continuous phase ($\vec{v_{\text{cp}}}$) and the dispersed phase ($\vec{v_{\text{dp}}}$). Therein,

(2) $$\alpha =\frac{3}{4}C_{\text{d}}\frac{\rho_{\text{cp}}\Psi_{\text{cp}}\Psi_{\text{dp}}\left|\vec{v_{\text{cp}}}-\vec{v_{\text{dp}}}\right|}{d_{\text{dp}}\xi }$$

where the density and volume fraction of the continuous phase are $\rho_{\text{cp}}$ and $\Psi_{\text{cp}}$, respectively, and the volume fraction of the dispersed phase is $\Psi_{\text{dp}}$. The drag coefficient $C_{d}$ on a single sphere is related to the Reynolds number ($Re_{\text{p}}$), as per the well-known Schiller–Naumann drag model (Naumann and Schiller, 1935):

(3) $$C_{\text{d}}=\left\{\begin{array}{ll} \frac{24}{Re_{\text{p}}}\left(1+0.15Re_{\text{p}}^{0.687}\right), & \text{if}\;Re_{\text{p}}>1000\\ 0.44, & \text{otherwise} \end{array}\right.$$

(4) $$Re_{\text{p}}=\frac{\rho_{\text{cp}}d_{\text{dp}}\xi \left|\vec{v_{\text{cp}}}-\vec{v_{\text{dp}}}\right|}{\eta_{\text{cp}}}$$

where $d_{\text{dp}}$ is the diameter of blood cells in the dispersed phase, $\eta_{\text{cp}}$ is the dynamic viscosity of the continuous phase, and ξ is the dynamic shape factor. More drag is experienced by agglomerating non-spherical particles in a fluid (Iimura and Higashitani, 2005). The dynamic shape factor of RBCs was used to describe the effect of RBC agglomeration as

(5) $$\xi =\left\{\begin{array}{ll} 1.5{\left[1+(\tau \dot{\gamma }{)}^{2}\right]}^{0.058997}, & \text{if}\;\dot{\gamma }\leq 300\\ 1, & \text{otherwise} \end{array}\right.$$

Similarly, the shape factor for WBCs is assumed to be 1, as they do not undergo agglomeration like RBCs. Two external forces, viz. virtual mass and lift force, were neglected because they are minimal in comparison to drag force. The numerical solution method makes use of the implicit, unstructured mesh of finite volume elements. Note that in our model, plasma has been classified as the primary phase while RBCs and WBCs are classified as the secondary phases.

The simulations employed a segregate solver, with SIMPLEC pressure–velocity coupling and first order upwind spatial discretization. Solution convergence was monitored by minimizing the residuals for the mass continuity and velocity components. For pressure gradient-driven laminar flow solutions, the typical execution time was 1–2 days for 20,000 iterations with 0.0001 s time-step sizes using parallel computations with four processors operating at 3.1 GHz on Xeon nodes. The pressure and velocity data at the entry point to the tumor interior (around the fenestra opening) adjacent to the blood vessel depression were collected from this solution in order to be used as input field parameters in the subsequent simulation of plasma perfusion inside the tumor region. The density and viscosity of the plasma (Nithiarasu, 2022) were set at 1030 kg/m^3^ and 0.001 kg/(m·s), respectively. The density and viscosity of WBCs were determined to be 1080 kg/m^3^ and 0.011 kg/(m·s), respectively. On the other hand, the viscosity of RBCs is determined by our assigned functions and their density is set at 1100 kg/m^3^. The shape factor influences the phase interaction between plasma and RBCs (through our custom-made functions). The diameters of RBCs and WBCs (Schwartz and Conley, 2020) were set to 7 and 14 μm, respectively.

### 2.3 Simulation of plasma perfusion inside the tumor region

The mechanics of plasma transport through the fenestra is tracked as a viscous-laminar transient state flow. We obtained the numerical solution by employing the Eulerian multiphase model. It constitutes an advanced multiphase setup with high interaction between the continuous and dispersed phases. The solution is based on a single pressure shared by all phases, with continuity, momentum, and energy equations tracked for each (Afolabi and Lee, 2014). In the Eulerian model, drag coefficient functions are provided for several multiphase regimes. Models of drag coefficients based on local Reynolds numbers are widely used to represent phase coupling through inter-phase exchange terms. Herein, while simulating the transport physics inside the tumor, plasma is again modeled as the primary phase, whereas air is the secondary phase. Air is used to generate a “vacuum” inside the tumor region to extract an uncontaminated perfusion trend of plasma inside the tumor stroma and through the spaces in between the extracellular fiber bundles. The volume fraction of air is set to 1.0 during the initialization of simulation, meaning that at time 0 s, the entire tumor region is filled with air. The air will eventually be replaced by plasma as the simulation progresses.

The numerical solution’s convergence was again determined by minimizing the residuals of the mass and velocity components. For the pressure-gradient-driven laminar flow solutions, typical execution time was 3–4 h for 3000 iterations with 0.0001 s time-steps (for Model 1, Model 2, and Model 3), using 4-processor-based parallel computations operating at 3.1 GHz on Xeon nodes. Amongst additional physical parameters, the density and viscosity for air is assumed to be 1.225 kg/m^3^ and 1.7894×10^−5^ kg/(m·s), respectively. The diameter of air particle is set to 4.12×10^−4^ μm (Porterfield and Kruse, 1995). Note that our group has previously employed similar validated computational tools to replicate air and particle transport in complex respiratory physiology (e.g., see Basu (2021) and Basu et al. (2018, 2020)).

### 2.4 Enforced boundary conditions

During the simulation of the blood vessel region (see Section 2.2), the inlet pressure is set to 3325 Pa and is designated as the pressure inlet, the outlet pressure (Zhao et al., 2007) is set to 2128 Pa and is designated as the pressure outlet, and the fiber bundle surfaces (represented by the circular holes in the geometry shown, e.g., in Fig. 2(d)) have no-slip boundary condition. Further, at the blood vessel outlet, the back flow volume fraction is set to 0 for WBCs and 1 for RBCs. During the simulation of the tumor region, the inlet pressure for the mixed phases is set to the pressure values obtained from the blood vessel simulation, and the velocity is also set to the values obtained from the blood vessel simulation via user-defined commands. As a pressure outlet boundary condition, the air outlet pressure is set at 2780 Pa, which is extrapolated from an earlier study (Wu et al., 2013), and our extrapolated value agrees with the high interstitial pressure findings (Heldin et al., 2004; Sven and Josipa, 2007; Stylianopoulos et al., 2018) inside the tumor region.

## 3 Results

### 3.1 Simulated transport parameters inside the blood vessel

**Fig. 4:**
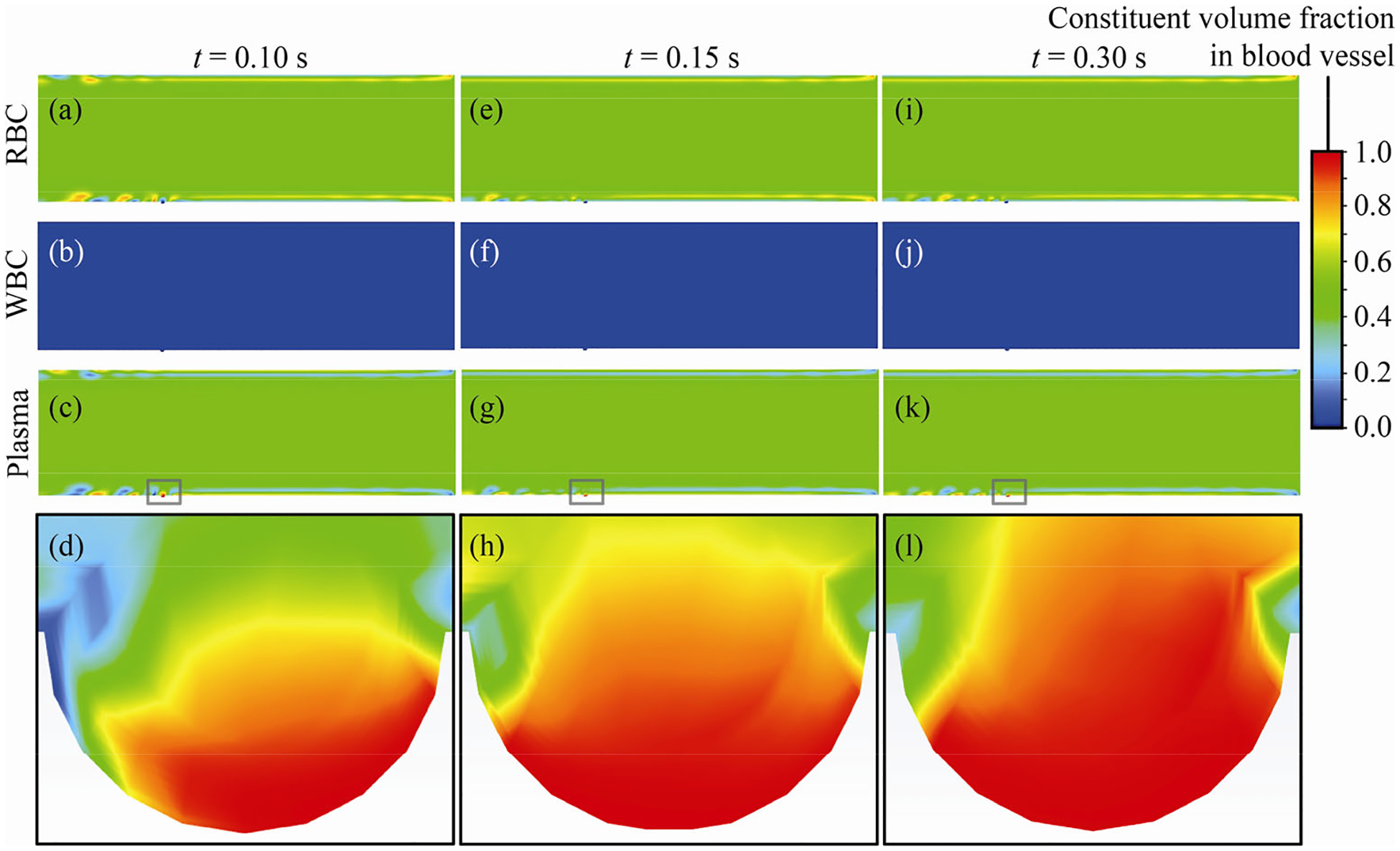
(a–c) Blood flow scenario with 3 phases inside the blood vessel with depression; the three phases are: RBCs, WBCs, and plasma. (a–d) Volume fraction color maps corresponding to the 3 modeled phases when flow time is 0.10 s; (e–h) corresponding states at 0.15 s; and (i–l) states at 0.30 s. (d), (h), and (l) demonstrate the spatio-temporal perturbations in the plasma volume fractions and are respectively zooming over the artificial circular depression in (c), (g), and (k), which mark the entry to the fenestra opening in the biomimetic model. Color scheme: deep green zone volume fraction range 0.50–0.58, light green zone volume fraction range 0.35–0.49, and dark blue zone volume fraction range 0–0.10.

Analysis of the volume fraction trends from the blood vessel simulation reveals that plasma has a constituent volume fraction greater than 0.50 and less than 0.60, RBCs have a constituent volume fraction greater than 0.40 and less than 0.50, and WBCs have a constituent volume fraction less than 0.10. Figure 4 depicts these scenarios for different flow time intervals of 0.10, 0.15, and 0.30 s. These findings are consistent with earlier reports (e.g., see McWhirter et al. (2012)). Also, the resulting plasma pressure and velocity distribution at the fenestra opening provided the inputs for simulating intratumoral plasma uptake, as discussed next.

### 3.2 Perfusion of plasma into the tumor vasculature

**Fig. 5:**
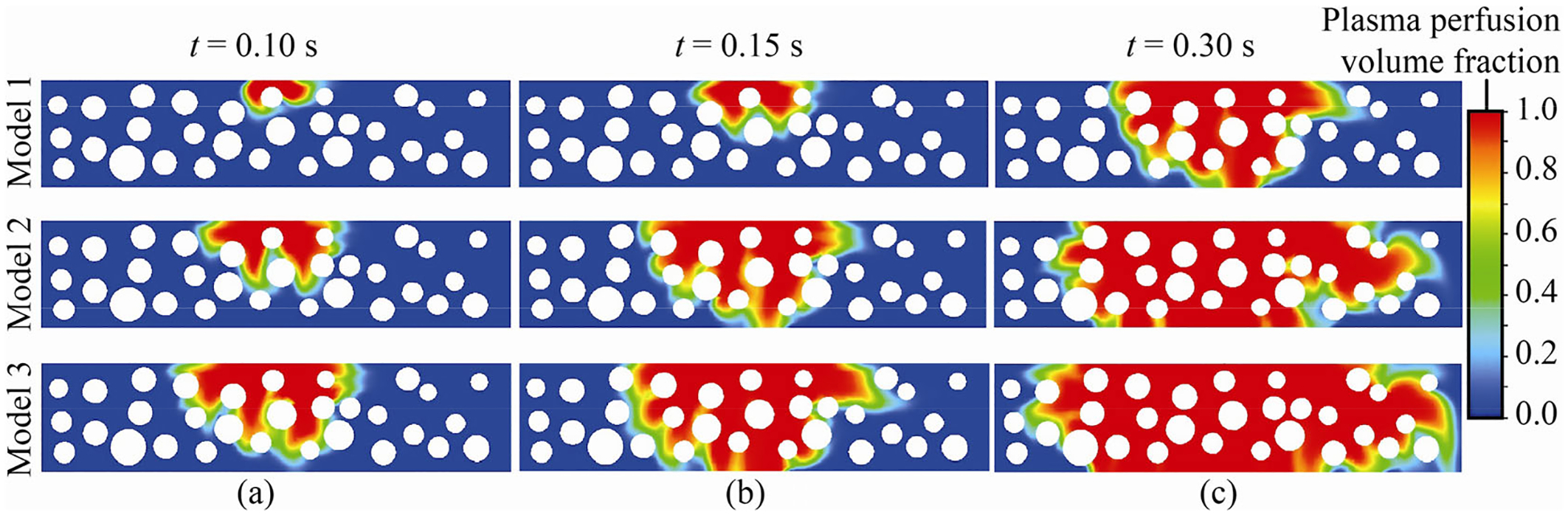
Plasma leakiness trend into the tumor domain for three distinct flow times. Model 1 has a fenestra diameter of 0.1 μm, Model 2 has a fenestra diameter of 0.3 μm, and Model 3 has a fenestra diameter of 0.5 μm. The blue zoned area is dominated by air, while the red and green zoned areas denote the plasma leakiness trend within the tumor region.

Eulerian two-phase simulations with plasma as the primary phase and air as the secondary phase quantified the perfusion trends in the tumor extracellular space. The pressure and velocity measurements obtained at each time step of the simulations in the main blood vessel are used as transient inlet boundary conditions for the tumor region. When the diameter of the fenestra remains constant, we can observe the following: in the first row (Fig. 5), the fenestra diameter is 0.1 μm, but the flow time increases from left to right, resulting in increased plasma percolation (indicated by the red zones in Fig. 5). When we examine the first column of Fig. 5, we notice a similar trend of plasma percolation, where the diameter of the fenestra varies from 0.1 to 0.3 μm, and then to 0.5 μm, but the flow duration of 0.10 s remains constant. Thus, leakiness of the plasma increases progressively as the fenestra dimension and flow time increase. We can observe that at 0.30 s flow time (Model 2, Model 3), the ratio of plasma to air is larger than one (Table 1), indicating that plasma has approached its percolation limit and has mostly replaced air in the tumor.

### 3.3 Quantifying the relation between the fenestra diameters and the rates of diffusion

By analyzing the spatio-temporal plasma perfusion trends represented through the color palette data in Table 1, we can infer that for the same flow time, the combined area of red and green zones (Fig. 5) increases relative to the blue zone area as the fenestra diameter increases. The combined area of red and green represents plasma percolation.

We show next how the size of the opening of the fenestra quantitatively affects the rate at which plasma moves through the tumor. At any specific time stamp during the plasma perfusion process, let there be a mathematical idealization whereby

(6) $$\frac{{\left(A_{\text{R}+\text{G}}\right)}_{\text{at}D_{i}}}{{\left(A_{\text{R}+\text{G}}\right)}_{\text{at}D_{k}}}={\left(\frac{D_{i}}{D_{k}}\right)}^{x_{n}}$$

Here, $A_{\text{R}+\text{G}}$ represents the combined area of red and green regions (Table 1) in the maps of Fig. 5, and D indicates the diameter of fenestra, i and k correspond to the different diameter values, and $x_{n}$ indicates the power of the fenestra diameter ratio for a specific flow time.

Therefore, as a representative case, for flow time = 0.1 s we can write:

(7) $$\frac{{\left(A_{\text{R}+\text{G}}\right)}_{{\text{at}D}_{0.3}}}{{\left(A_{\text{R}+\text{G}}\right)}_{{\text{at}D}_{0.1}}}={\left(\frac{D_{0.3}}{D_{0.1}}\right)}^{x_{n}}\Rightarrow \frac{0.087}{0.021}={\left(\frac{0.3}{0.1}\right)}^{x_{1}}\Rightarrow x_{1}=1.29$$

Table 2 depicts the power ratio estimates (derived following the same strategy as above) for different flow times (e.g., 0.15 s, 0.30 s) and diameter ratios. Note here that the technique to implement scaling on the diameter ratio assists in normalizing the effect of fenestra opening sizes on the diffusion trends.

Considering the data from the first and last columns of Table 2, the slope is found to be 4 between (0.10, 1.09) and (0.15, 1.29), and 0.67 between (0.15, 1.29) and (0.30, 1.39). As can be seen, the slope is decreasing, indicating that the rate of diffusion is decreasing in lockstep with the increase in diffusion distance as flow time increases. However, the plasma components continue to spatio-temporally disperse away from the fenestra opening into the tumor. This scenario lends credence to Fick’s law (Bouzin and Feron, 2007; West, 2012), according to which the rate of diffusion is inversely proportional to the distance over which it occurs, and is mathematically:

(8) $$R=\frac{DA\delta P}{d}$$

Here, R is the rate of diffusion, d is the distance over which diffusion takes place, A is the area of diffusion coverage, δP indicates the pressure difference driving the flow, and D is diffusion coefficient. So R∝1/d (other factors remaining invariant), which implies that the rate of perfusion will decrease if the diffusion distance increases as the plasma move further from the fenestra opening into the tumor stroma.

## 4 Discussion

- On the geometric restrictions to preserve physical realism of the transport mechanics

The diameter of the rounded depression in the blood vessel geometry and its location marking the fenestra opening are chosen systematically such that it remains significantly wider (> 10-fold) than the maximum diameter of the fenestra (0.5 μm), implying that the fenestra should lie within the depression. Additionally, the depression’s depth is 2.6 μm, which is greater (> 8-fold) than the fenestra’s streamwise length of 0.3 μm. However, these values were chosen arbitrarily in order to simplify the biomimetic model. The test geometry was divided into two segments for the primary reason of algorithmically restricting the entry of larger particles (e.g., RBCs whose diameters are appreciably larger compared to the typical fenestra openings and endothelial clefts) into the tumor via the modeled fenestra. Also, the numerical scheme has employed user-defined functions built based on earlier published studies, e.g., Jung and Hassanein (2008) and Jung et al. (2006), and has been compared for validation against the experimental results from Karino and Goldsmith (1977).

- On the limitations in the simulated blood transport model

The luminal surface of the endothelial cells contain patches of anionic layers, called the glycocalyx (Fig. 2(f)). In our model, we have not considered the resulting electrohydrodynamic effects that may emerge in the mean transport, and we expect to address this in our future work. In this context, see Koumoutsakos et al. (2013) for a rich review of the open fluid mechanics questions associated with diagnosis, predictive modeling, and therapy of cancer.

- On the limitations of plasma perfusion simulations inside the tumor region

In our reduced order model, we did not consider plasma transportation within the entire volume of the tumor; rather the focus was on a small portion of tumor comprising the connective tissue region encircling the necrotic core in order to quantify the leakiness trend of plasma within the tumor region. The outcome is summarized in Table 1. Also, the dynamics of plasma perfusion inside the tumor stroma is dominated by the diffusive length scale. The latter is analogous to the streamwise length scale locally, while the fluid layers move around and diffuse through the spaces in between the rounded cross-sections of the fiber bundles. With flow feature variations in the streamwise direction generally much lower than those in the wall normal direction, the simulated system is markedly devoid of strong boundary layer formations along the fiber walls (Fig. 5), which otherwise were enforced with no-slip boundary condition.

- On the time scales in the computations and their relevance in a clinical perspective

While the time scales reported, e.g., in Fig. 5, are fractions of seconds, it should be noted that these constitute the computed time intervals. The realistic time periods for comparable percolation in a real tumor, with an underlying medium of cellular constituents present in between the fiber bundles, could be greater. Careful comparison of the *in silico* transport projections with experimental measurements of intratumoral uptake in real tumors can generate the appropriate scaling factors by which the reported computational time intervals should be multiplied to obtain clinically relevant temporal projections of diffusion. Evaluation of such time scales is also the key toward assessing the hypoxia effects (Duffy et al., 2003) near the necrotic core of a malignant tumor.

- On the topological simplicity of the modeled blood vessel

The profile of the wall boundaries (comprising the endothelial lining) of the blood vessel in our 2D biomimetic model has been assumed to be straight, e.g., see the top and bottom walls in Fig. 2(a). However, in reality the blood vessel architecture could often be significantly tortuous and may present random curvatures (e.g., Fig. 2(g)). The presented *in silico* modeling framework can hence be enhanced by structuring vessel geometries directly from the tumor vasculature imaging data.

- On extending the modeling framework through CT (computed tomography) scanning of real tumors

Human pancreatic cancer cells were orthotopically injected into the pancreas of nude mice to establish human pancreatic tumors. To assess a realistic scenario wherein our modeling strategies can find applicability, pancreatic orthotopic tissues from three nude mice were collected and fixed for 24 h in formaldehyde. Paraffin-embedded 5 μm thick sections of tumor tissues were prepared. Sections were deparaffinized with Histo-Clear and ethanol. Then tissue slides were rehydrated and stained with hematoxylin for 5 min. The slides were subsequently washed with distilled water, soaked in 95% ethanol for 30 s, and stained with eosin for 1 min. Next, they were dehydrated with 100% ethanol for 1 min, washed in xylene and mounted with a coverslip using a Hardset Mounting Medium. Finally, slides were visualized using a Zeiss inverted Axio Observer Z1 microscope.

**Fig. 6:**
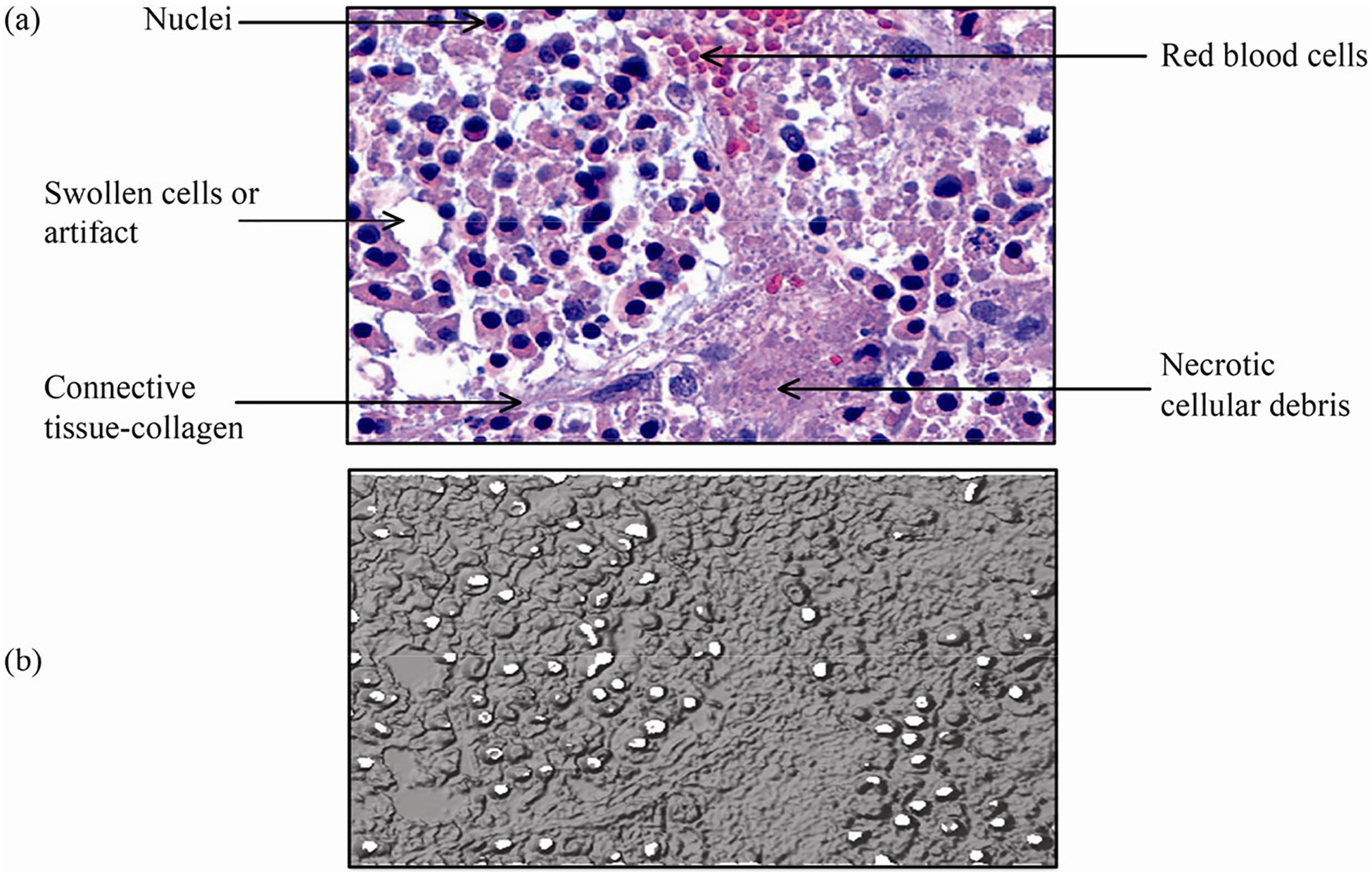
(a) Representative image from H&E (hematoxylin and eosin) staining done to identify overall architecture (shape, pattern, and structure of cells) of human pancreatic orthotopic tissue. The staining was performed on pancreatic tissue from three different samples. (b) STL (stereolithography) reconstruction of (a).

To determine whether our STL (stereolithography) imaging reconstruction can accurately replicate the overall architecture of tumor tissue (thus easing the first step of the computational model generation process), we compared a stained human pancreatic orthotopic cancer tissue sample (with hematoxylin and eosin, or H&E staining) to the STL version of the same section of the stained cancer tissue 365 sample. The H&E stain technique, the most commonly used modality in medical diagnosis, especially by pathologists for cancer diagnosis, provides information on overall cell structure, patterns, and shapes in a tissue sample. We observed that the overall architecture as well as the microanatomy of the stained tumor tissue section (Fig. 6(a)) is nearly identical to the tissue scan from the STL image (Fig. 6(b)). For example, the necrotic areas in both images (Fig. 6) were nearly identical in size and pattern. Furthermore, the STL geometry accurately pinpointed the cells undergoing apoptosis and swelling (Fig. 6). We also noted that the STL reconstruction recognized the cellular nuclei within the tumor samples. Collectively, this preliminary assessment demonstrated that our *in silico* methods can accurately replicate the overall architecture of human pancreatic cancer orthotopic tissue samples.

## 5 Conclusions

Diffusive transport into solid tumors is restricted due to their dense fibrous extracellular structure, whereas convection is difficult owing to a lack of blood and lymphatic movements. The complexity of fluidic transport inside the tumor microenvironment comes with unanswered questions with potentially significant translational implications. For this study, we have built a 2D biomimetic model of a blood vessel with an adhering reduced-order tumor extracellular domain with fenestra openings to the vasculature. In our CFD-based work, plasma percolation is found to be proportional to the leakiness of the fenestra aperture and to the increase in flow duration tracked. The plasma percolation, however, decayed with increasing intratumoral diffusion distance from the blood vessel opening (fenestra). Although we conducted our study on a small chunk of tumor, the study’s outcome indicates that the *in silico* technique bears the potential for implementation across the entire tumor spheroid. This CFD transportation model can also be applied to the real tumor vasculature; for this purpose, we have already stained real mice tumors and constructed an STL 2D domain of the tumor architecture.

In summary, the derived intratumoral plasma penetration levels are clearly impacted by tumor leakiness (modulated by varying the fenestra openings) and diffusion distances. This work can have key implications for *in silico* diagnosis and drug delivery estimates for hard-to-reach tumor types using first-principles, reduced-order computational models built from high-resolution medical imaging data.

## Figures and Tables

**Table 1:** Color palette analysis of Fig. 5: A spatio-temporal representation of plasma perfusion trend into the tumor extracellular space, represented by the ratio of the summation of red (R) and green (G) domains over the blue (B) region

Flow time (s)	0.10	0.15	0.3
Model 1	(R+G):B = 0.021:1	(R+G):B = 0.024:1	(R+G):B = 0.42:1
Model 2	(R+G):B = 0.087:1	(R+G):B = 0.167:1	(R+G):B = 4.10:1
Model 3	(R+G):B = 0.141:1	(R+G):B = 0.219:1	(R+G):B = 4.65:1

**Table 2:** Ratio of the power for the different fenestra diameter ratios to estimate perfusion trend comparisons over different flow time

Flow time (s)	Power (x) for $\frac{D_{0.3}}{D_{0.1}}$	Power (y) for $\frac{D_{0.5}}{D_{0.1}}$	$\frac{x}{y}$
0.10	1.29	1.18	1.09
0.15	1.77	1.37	1.29
0.30	2.07	1.49	1.39

## Data Availability

The digitized biomimetic geometry and the simulation data sets are available on-request from the corresponding author.
